# Supplementary material for: Brain Perfusion Alterations Induced by Standalone and Combined Non-Invasive Brain Stimulation over the Dorsolateral Prefrontal Cortex
Source: Biomedicines. 2022 Sep 27;10(10):2410. doi: 10.3390/biomedicines10102410 (PMC9598449; doi:10.3390/biomedicines10102410)
Supplement: Supplementary file 1 [file biomedicines-10-02410-s001.zip › biomedicines-1909082-supplementary.pdf]

**Supplementary Material**

**Figure S1. Prefrontal cortex based on the Sallet Atlas (Sallet et al. 2013).**

**Figure S2. Neuronavigated coordinates overlapping Brodmann areas 9/46D and 9/46V.**

**Figure S3. Flowchart.**

**Table S1. Demographic and baseline measures.**

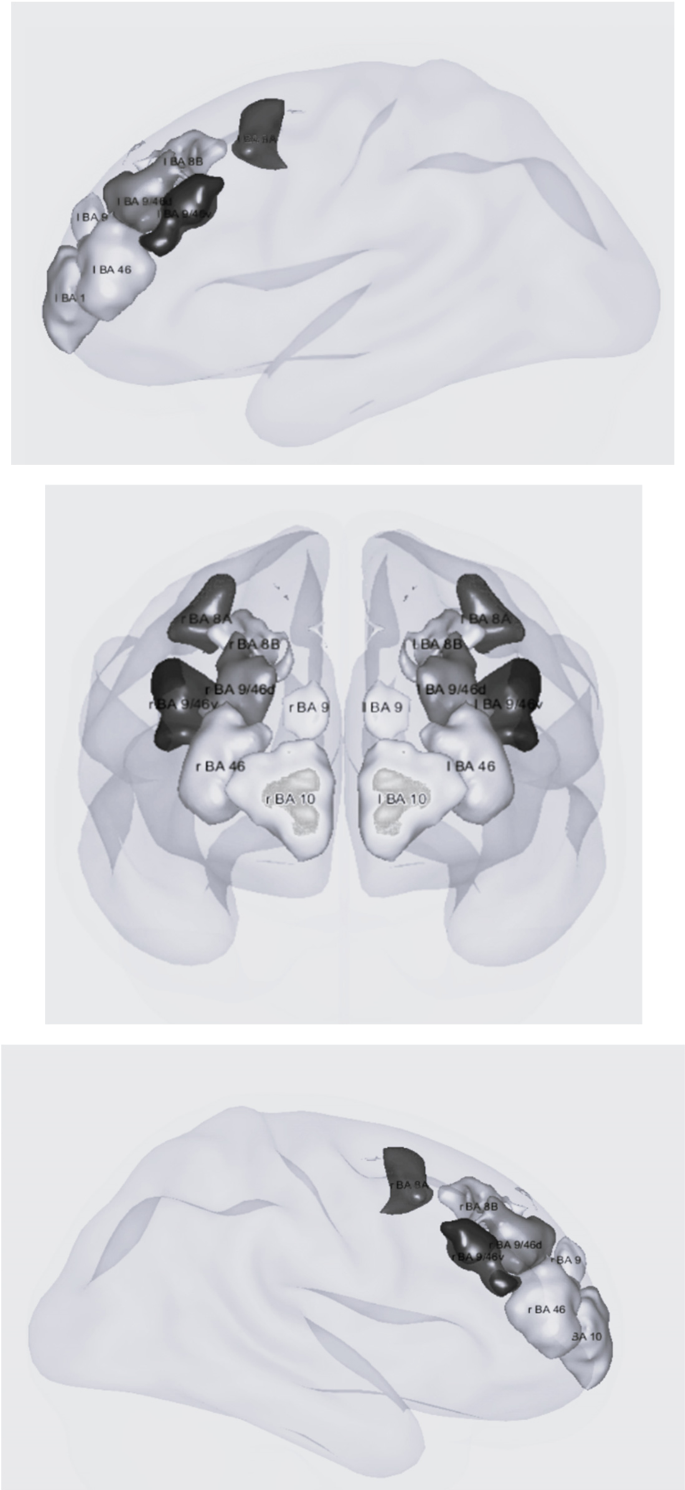


**Figure S1. Prefrontal cortex based on the Sallet Atlas (Sallet et al. 2013).** Seven dorsolateral prefrontal cortex sub-regions used in our analyses, corresponding to: brodmann area (BA) 9, BA 10, BA 9/46D and BA 9/46V, BA 46, BA 8A and BA 8B.

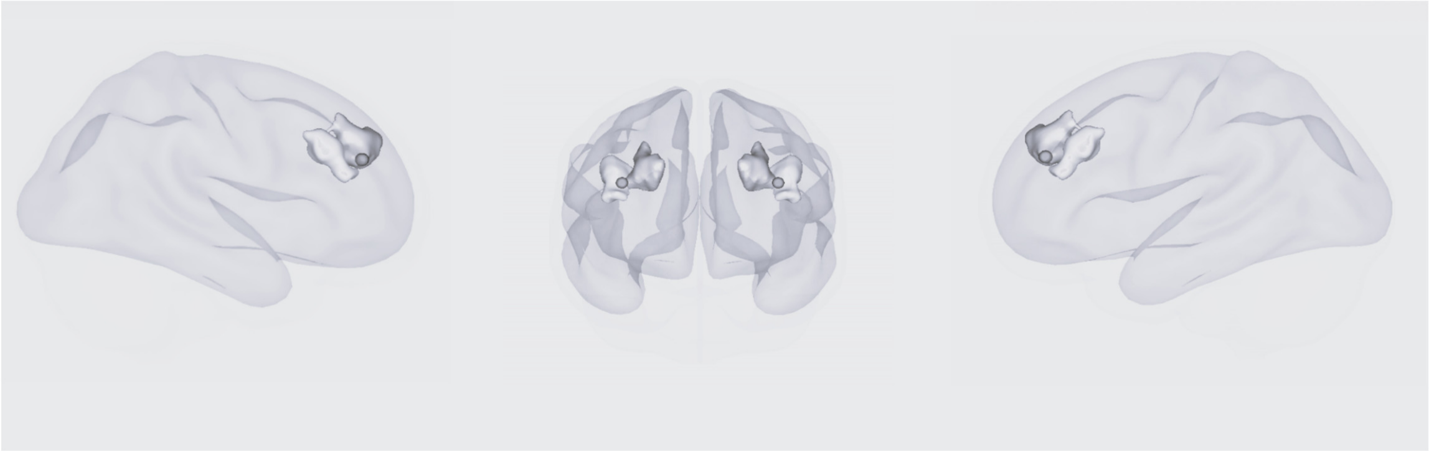

**Figure S2. Neuronavigated coordinates overlapping Brodmann areas 9/46D and 9/46V.** Neuronavigated coordinates were based on the MNI152 stereotaxic atlas (coordinates,-38, +44, +26 and +38, +44, +26). Note: The circular areas correspond to the left and right MNI coordinates (targeted DLPFC), while the light gray corresponds to the BA 9/46V region and the dark gray corresponds to the BA 9/46D region.

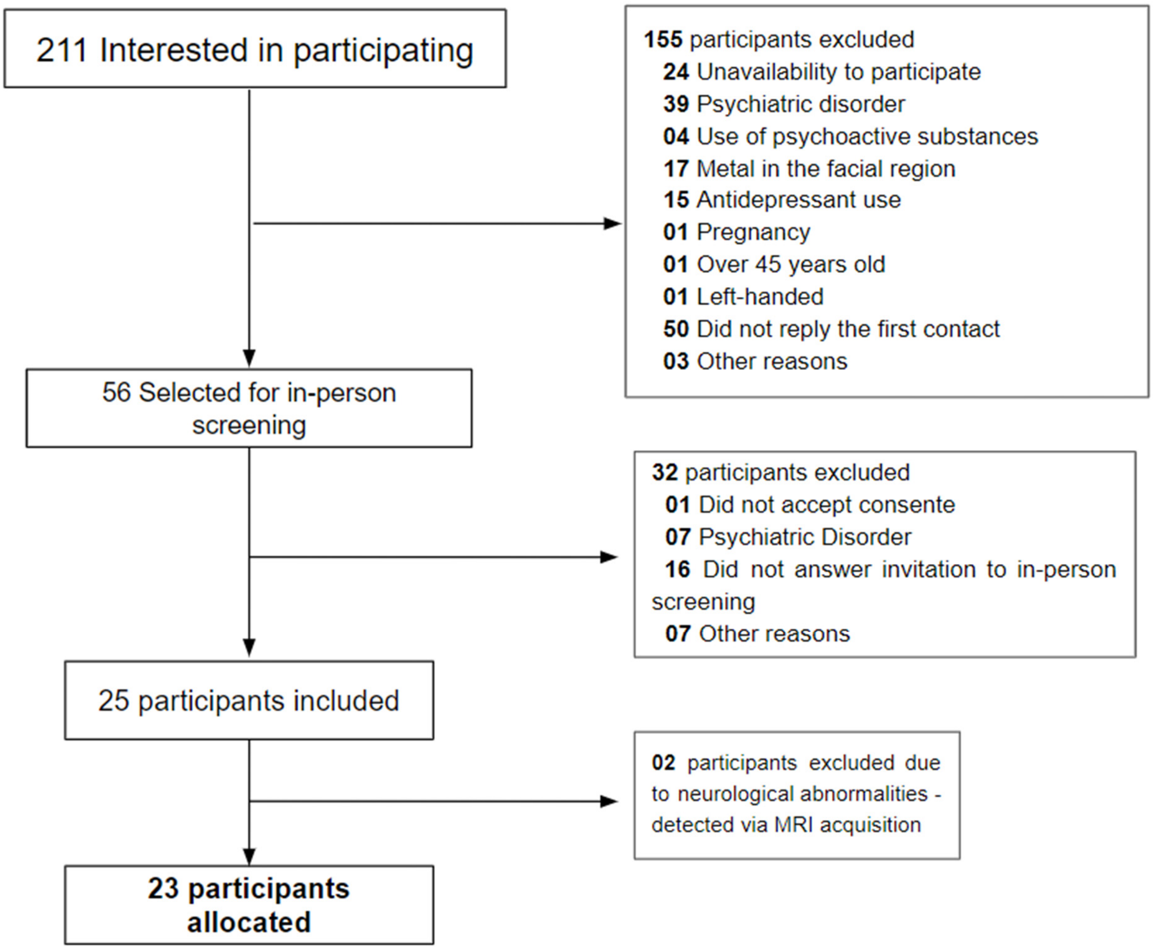


**Figure S3. Flowchart.**

**Table S1. Demographic and baseline measures.**

| Right sACC | | | |
|---|---|---|---|
| **Predictors** | **Estimates** | **95% CI** | **p** |
| iTBS | -9.15 | -21.65 – 3.34 | 0.149 |
| tDCS | -7.68 | -20.46 – 5.09 | 0.235 |
| sex | 8.63 | -14.02 – 31.28 | 0.45 |
| age | -0.24 | -1.69 – 1.22 | 0.748 |
| HDRS | 2.51 | -1.78 – 6.80 | 0.248 |
| PA | -0.04 | -1.14 – 1.07 | 0.948 |
| NA | -0.35 | -2.21 – 1.50 | 0.706 |
| ***iTBS*tDCS*** | ***19.82*** | ***2.23 – 37.41*** | ***0.028*** |

| Left sACC | | | |
|---|---|---|---|
| **Predictors** | **Estimates** | **95% CI** | **p** |
| iTBS | 0.66 | -0.89 – 2.20 | 0.4 |
| tDCS | -0.03 | -1.54 – 1.48 | 0.971 |
| sex | 1.27 | -1.13 – 3.67 | 0.294 |
| age | 0 | -0.15 – 0.15 | 0.996 |
| HDRS | 0.21 | -0.30 – 0.72 | 0.41 |
| PA | 0.08 | -0.05 – 0.21 | 0.236 |
| NA | -0.1 | -0.32 – 0.11 | 0.353 |
| iTBS*tDCS | -0.48 | -2.61 – 1.65 | 0.656 |

| Right ROI 9/46V, 9/46D | | | |
|---|---|---|---|
| **Predictors** | **Estimates** | **95% CI** | **p** |
| iTBS | 1.85 | -21.75 – 25.46 | 0.876 |
| tDCS | 15.86 | -7.27 – 38.98 | 0.176 |
| sex | -16.47 | -54.35 – 21.40 | 0.389 |
| age | -3.06 | -5.49 – -0.62 | 0.014 |
| HDRS | -0.43 | -8.28 – 7.42 | 0.913 |
| PA | 0.45 | -1.54 – 2.44 | 0.651 |
| NA | -1.71 | -5.04 – 1.62 | 0.31 |
| iTBS*tDCS | -3.41 | -35.95 – 29.13 | 0.835 |

| Left sACC | | | |
|---|---|---|---|
| **Predictors** | **Estimates** | **95% CI** | **p** |
| iTBS | 0.66 | -0.89 – 2.20 | 0.4 |
| tDCS | -0.03 | -1.54 – 1.48 | 0.971 |
| sex | 1.27 | -1.13 – 3.67 | 0.294 |
| age | 0 | -0.15 – 0.15 | 0.996 |
| HDRS | 0.21 | -0.30 – 0.72 | 0.41 |
| PA | 0.08 | -0.05 – 0.21 | 0.236 |
| NA | -0.1 | -0.32 – 0.11 | 0.353 |
| iTBS*tDCS | -0.48 | -2.61 – 1.65 | 0.656 |

*: interaction between iTBS and tDCS

**Reference**


1. Sallet, J.; Mars, R.; Noonan, M.P.; Neubert, F.-X.; Jbabdi, S.; O'Reilly, J.; Filippini, N.; Thomas, A.; Rushworth, M. The Organization of Dorsal Frontal Cortex in Humans and Macaques. *J. Neurosci.* **2013**, *33*, 12255–12274. https://doi.org/10.1523/jneurosci.5108-12.2013.
